# Retraction Note: Bone mesenchymal stem cell-derived exosomal microRNA-7-5p inhibits progression of acute myeloid leukemia by targeting *OSBPL11*

**DOI:** 10.1186/s12951-025-03124-4

**Published:** 2025-01-18

**Authors:** Duanfeng Jiang, Xin Wu, Xiaoying Sun, Wei Tan, Xin Dai, Youbang Xie, Ashuai Du, Qiangqiang Zhao

**Affiliations:** 1 Department of Hematology, Second Affiliated Hospital of Hainan Medical College, Haikou, 570311 People’s Republic of China; 2 Department of Hematology, The Third Xiangya Hospital, Central South University, Changsha, 410013 People’s Republic of China; 3 Department of Orthopedics, Third Xiangya Hospital, Central South University, Changsha, 410013 People’s Republic of China; 4 Nursing School, Soochow University, Suzhou, 215000 People’s Republic of China; 5 Department of Emergency, The Qinghai Provincial People’s Hospital, Xining, 810007 People’s Republic of China; 6 Department of Hematology, The Qinghai Provincial People’s Hospital, Xining, 810007 People’s Republic of China; 7 Department of Infectious Diseases, Guizhou Provincial People’s Hospital, Guiyang, 550002 People’s Republic of China; 8 Department of Blood Transfusion, The Third Xiangya Hospital, Central South University, Changsha, 410013 People’s Republic of China

**Retraction Note: Journal of Nanobiotechnology (2022) 20:29**

10.1186/s12951-021-01206-7

The Editor-in-Chief has retracted this article because of concerns regarding the figures presented in this work. These concerns call into question the article’s overall scientific soundness. An investigation conducted after its publication discovered the following issues:

• A portion of panel MOLM13, Exo-Nc-mimics in Fig. 6D appears to overlap with a portion of panel MOLM13, Exo-Nc-inhibitor in the same figure;

• A portion of panel MOLM13, Exo-miR-7-5p-inhibitor in Fig. 6D appears to overlap with a portion of panel MOLM13, c in Fig. 4D.

The Authors provided some raw data on request from the Publisher. However, this was insufficient to satisfy the concerns raised. The Editor-in-Chief no longer has confidence in the integrity of the research presented in this article.

The authors have not stated whether they agree or disagree with this retraction.

